# Transthyretin provides trophic support via megalin by promoting neurite outgrowth and neuroprotection in cerebral ischemia

**DOI:** 10.1038/cdd.2016.64

**Published:** 2016-08-12

**Authors:** J R Gomes, RS Nogueira, M Vieira, SD Santos, J P Ferraz-Nogueira, J B Relvas, M J Saraiva

**Affiliations:** 1 Instituto de Investigação e Inovação em Saúde (I3S), University of Porto, Porto, Portugal; 2 Molecular Neurobiology Unit, IBMC- Institute for Molecular and Cell Biology, University of Porto, Porto, Portugal; 3 Glial Cell Biology Unit, IBMC- Institute for Molecular and Cell Biology, University of Porto, Porto, Portugal; 4 ICBAS-Instituto de Ciências Biomédicas Abel Salazar, Universidade do Porto, Rua de Jorge Viterbo Ferreira nº 228, 4050-313 Porto, Portugal

## Abstract

Transthyretin (TTR) is a protein whose function has been associated to binding and distribution of thyroid hormones in the body and brain. However, little is known regarding the downstream signaling pathways triggered by wild-type TTR in the CNS either in neuroprotection of cerebral ischemia or in physiological conditions. In this study, we investigated how TTR affects hippocampal neurons in physiologic/pathologic conditions. Recombinant TTR significantly boosted neurite outgrowth in mice hippocampal neurons, both in number and length, independently of its ligands. This TTR neuritogenic activity is mediated by the megalin receptor and is lost in megalin-deficient neurons. We also found that TTR activates the mitogen-activated protein kinase (MAPK) pathways (ERK1/2) and Akt through Src, leading to the phosphorylation of transcription factor CREB. In addition, TTR promoted a transient rise in intracellular calcium through NMDA receptors, in a Src/megalin-dependent manner. Moreover, under excitotoxic conditions, TTR stimulation rescued cell death and neurite loss in TTR KO hippocampal neurons, which are more sensitive to excitotoxic degeneration than WT neurons, in a megalin-dependent manner. CREB was also activated by TTR under excitotoxic conditions, contributing to changes in the balance between Bcl2 protein family members, toward anti-apoptotic proteins (Bcl2/BclXL *versus* Bax). Finally, we clarify that TTR KO mice subjected to pMCAO have larger infarcts than WT mice, because of TTR and megalin neuronal downregulation. Our results indicate that TTR might be regarded as a neurotrophic factor, because it stimulates neurite outgrowth under physiological conditions, and promotes neuroprotection in ischemic conditions.

TTR is synthesized in the liver (blood source) and choroid plexus (CSF-cerebrospinal fluid source). The physiological functions of TTR are the transport of thyroxine and retinol, through RBP (retinol-binding protein). More recently, TTR was described to have roles independent of its ligands, from neuroprotection in Alzheimer and schizophrenia, to the involvement in memory and learning.^1^ It was also associated with nerve regeneration in the peripheral nervous system (PNS).^2^ We have previously shown that TTR has a neuroprotective effect in focal cerebral ischemia in the central nervous system (CNS).^3^ Several other studies pointed in the same direction: (i) TTR can be a good predictor for young patients with stroke, because patients have worse clinical prognosis if they exhibit decreased serum TTR;^4^ (ii) smaller incidence of stroke in women, owing to neuroprotective action of sex steroids, which upregulate TTR in CSF;^5^ (iii) in a *C. elegans* model, a TTR-like protein was determinant in the recognition of apoptotic cells by phagocytes.^6^

TTR binds megalin (LRP-2),^7, 8^ RAGE^9^ (receptor for advanced glycation end products) and IGF-IR (insulin-like growth factor 1 receptor).^10^ Megalin has been mainly studied in kidney, but its function in the CNS is poorly understood.^11^ It binds metallothionein, clusterin and apolipoprotein-E, which have neuroprotective roles.^12^ Megalin activates intracellular pathways, such as Erk1/2 and Akt, leading to the activation of transcription factor CREB.^13^

Ischemic brain injury is a leading cause of mortality in Western countries.^14^ Tissue plasminogen activator is the only approved therapy for acute non-hemorrhagic stroke. However, this therapeutic strategy can only be used in the first 4.5 h after symptoms onset, making it available for only 4–7% of these patients.^15^ Excitotoxicity mediated by overactivation of glutamate receptors is one the major events in cerebral ischemia, playing a key role in neuronal death. Despite failure of previous generation of drugs, the new generation of excitotoxicity inhibitors may succeed, for example, Tat-NR2B9c peptide, is neuroprotective in both animals and patients.^16, 17^ Excitotoxic mechanisms are characteristic of several other disorders.^18^

In the present study, we unraveled the molecular pathways involving TTR in CNS neurons both during physiologic and pathologic/ischemic conditions. We found that TTR promotes a robust neurite outgrowth response in neurons, through upregulation of intracellular calcium and MAPK pathways, triggered by its interaction with megalin. Moreover, TTR/megalin interaction was found to be determinant for neuronal survival and neurite preservation, both in excitotoxic conditions in neuronal cultures, and in a mouse model of permanent middle cerebral artery occlusion (pMCAO).

## Results

### TTR promotes neurite outgrowth in WT and TTR KO cultured hippocampal neurons through megalin

**Figure 1:**
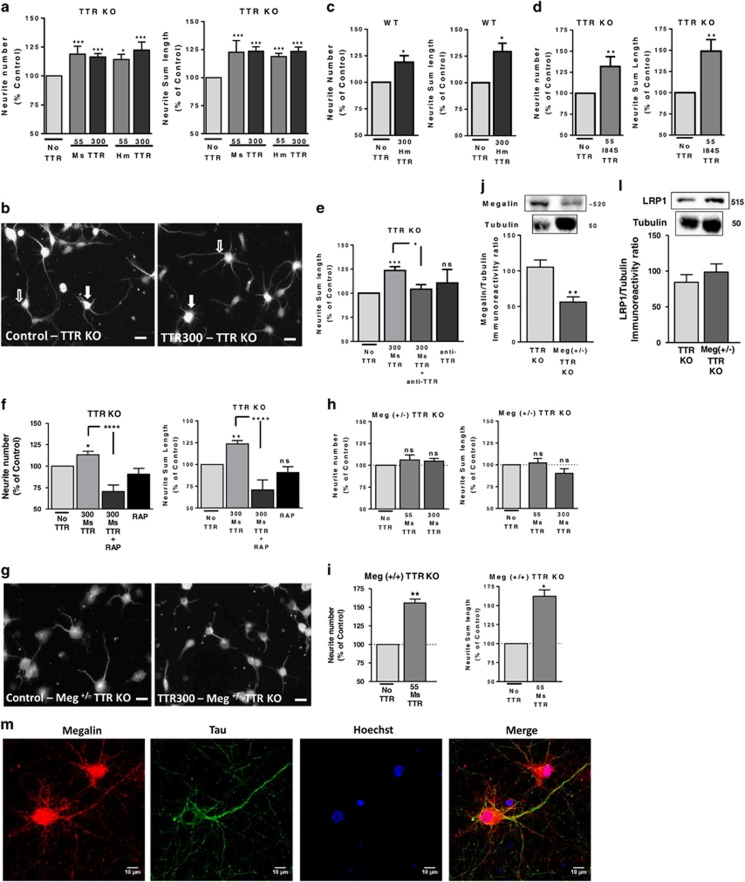
TTR promotes neurite outgrowth in WT and TTR KO cultured hippocampal neurons through megalin, independent of interaction with its ligands. (**a**) TTR KO cultured hippocampal neurons were stimulated with recombinant mouse and human TTR (55 and 300 *μ*g/ml), immediately after plating for 24 h, and stained with a MAP_2_ antibody. Neurite number and neurite sum length were quantified with ImageJ software. The results are the average±S.E.M. of 3–10 independent experiments. (**b**) Representative MAP_2_ staining of TTR KO cultured hippocampal neurons stimulated with TTR. (**c**) WT cultured hippocampal neurons were also stimulated with recombinant human TTR (300 *μ*g/ml), immediately after plating, for 24 h. Neurite number and neurite sum length were quantified as described in (**a** and **b**). The results are the average±S.E.M. of five independent experiments. (**d**) Corresponds to the same stimulation of TTR KO hippocampal neurons with a recombinant mutant TTR (TTR I84S 55 *μ*g/ml)). The results are the average±S.E.M. of three to four independent experiments. In (**e**), the results (neurite sum length) in similar experiments using TTR KO cultured hippocampal neurons treated with mouse TTR with or without an antibody against TTR are shown. The results are the average±S.E.M. of three to seven independent experiments. (**f**) TTR KO cultured hippocampal neurons were stimulated with recombinant mouse TTR (300 *μ*g/ml) with or without RAP and stained with MAP_2_ to quantify neurite number and neurite sum length. The results are the average±S.E.M. of four to seven independent experiments. Megalin heterozygous TTR KO cultured hippocampal neurons were also stimulated with recombinant mouse TTR (55 and 300 *μ*g/ml) immediately after plating for 24 h. Neurite number and neurite sum length for megalin (+/−) TTR KO (**h**) and megalin (+/+) TTR KO littermates (**i**) were quantified as described in (**a** and **b**). The results are the average±S.E.M. of three to four independent experiments. (**g**) Representative MAP_2_ staining of megalin (+/−) TTR KO cultured hippocampal neurons stimulated with TTR. Megalin (**j**) and LRP1 (**l**) protein levels in TTR KO *versus* megalin heterozygous TTR KO hippocampal cultures were determined by western blot (*n*=10 and 5, respectively). (**m**) Representative megalin, tau, MAP_2_ and Hoechst 33342 staining of TTR KO cultured hippocampal neurons (7 DIV) of three independent experiments. Statistical analysis was performed using one-way ANOVA followed by Bonferroni's multiple comparison test performed for each condition or Student's unpaired *t*-test for two-groups only comparisons. ***P<0.001, **P<0.01, *P<0.05, n.s., not significant as compared with the control or as indicated. Scale bar in (**b** and **g**) correspond to 20 *μ*m and in (**m**) to 10 *μ*m

TTR was shown to promote neurite outgrowth in the PNS, mainly in dorsal root ganglia neurons,^2^ but the molecular signaling pathways involved are yet to be explored, as well as whether TTR has a similar role in the CNS. To determine whether TTR has neuritogenic activity in the CNS, cultured hippocampal neurons from TTR KO mice were stimulated with recombinant mouse or human TTR (to address its potential use as a neuroprotective target in humans) using two concentrations, 55 and 300 *μ*g/ml, that mimic the two pools of TTR in the body, CSF and sera, respectively.^19^ These conditions induced a rise in both neurite number (Figures 1a and b closed arrow) and neurite length, assessed by MAP_2_ immunocytochemistry (Figures 1a and b open arrow). The effect is dose-dependent and identical for human and mouse TTR. We observed the same neuritogenic activity of TTR in WT hippocampal neurons incubated with human TTR 300 *μ*g/ml (neurite number and length (Figure 1c)). To further confirm TTR as the protein responsible for these effects, we used an anti-TTR antibody, which blocked neurite outgrowth (Figure 1e). Moreover, when TTR KO neurons were stimulated with recombinant TTR variant (I84S), which has very low affinity for thyroxine and RBP ligands,^20, 21^ they had a similar increase in neurite number and outgrowth (Figure 1d) to WT TTR, proving that TTR neurogenic activity is not related to its ligands. In the PNS, TTR neuritogenic activity was shown to be megalin-mediated.^2^ To investigate whether this occurred in CNS neurons, we used RAP (receptor associated protein), an LRP antagonist,^22^ which blocks megalin–TTR interaction.^7^ RAP blocked TTR-induced increase in neurite number and total length (Figure 1f). However, because RAP is a broad LRP inhibitor, we prepared hippocampal neuronal cultures from megalin (+/−) TTR KO mice embryos, which have significant lower amounts of megalin than TTR KO cultures (Figure 1j). None of the TTR concentrations used promoted neurite outgrowth in these cultures (Figures 1g and h) in opposition with cultures from the littermates megalin (+/+) TTR KO (Figure 1i), indicating that TTR neuritogenic activity in the CNS is megalin-mediated. Both TTR concentrations are over the Kd determined for the megalin–TTR interaction (500 nM).^8^ Moreover, expression of LRP_1_, a megalin-related protein, was not altered in these megalin (+/−) TTR KO neuronal cultures (Figure 1l). We analyzed megalin expression in TTR KO cultured hippocampal neurons and observed that megalin is expressed in cell body and dendrites (not stained for tau), and to a lower extent in axons (colocalized with tau) (Figure 1m).

### Signaling pathways activated by TTR are megalin-dependent in cultured hippocampal neurons

**Figure 2:**
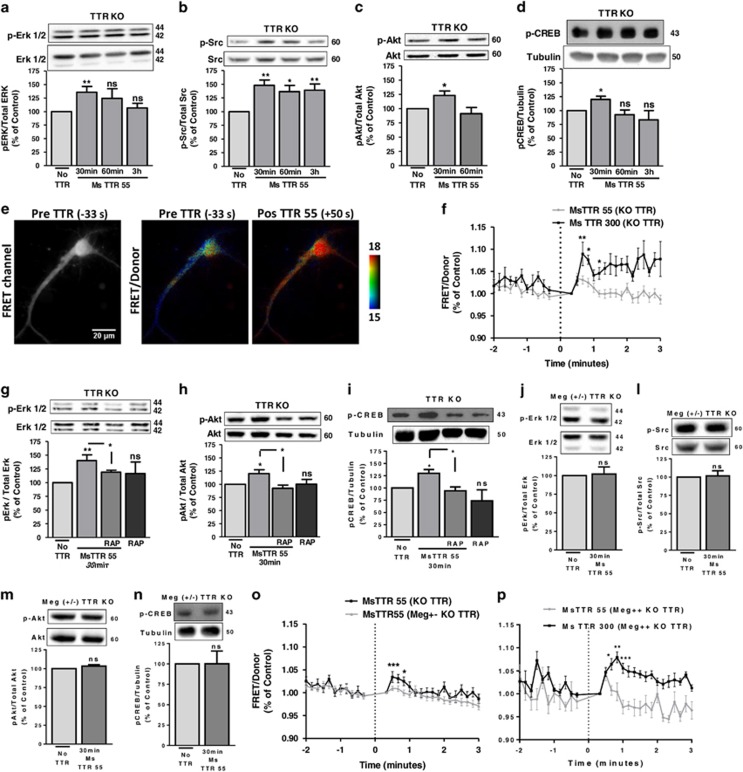
Signaling pathways activated by TTR are megalin-dependent. TTR signaling activity was assessed by analyzing the phosphorylation levels of ERK (**a**, *n*=3–14), SRC (Tyr416) (**b**, *n*=6–9), Akt (Ser473) (**c**, *n*=3–9) and CREB (Ser133) (**d**, *n*=3–14) following stimulation of TTR KO cultured hippocampal neurons (7 DIV) with recombinant mouse TTR (55 *μ*g/ml) for different time points. The results are the average±S.E.M. of 3–14 independent experiments. (**e**) FRET image and pseudocolor ratio images (FRET/Donor channels) of a representative TTR KO cultured hippocampal neuron expressing yellow Cameleon-Nano (YC-Nano15) stimulated with mouse TTR (55 *μ*g/ml). Scale bar represents 20 *μ*m and the FRET/Donor images were coded according to the indicated pseudocolor scale. (**f**) Time course of normalized FRET/Donor values in cell body of YC-Nano15-transfected TTR KO cultured hippocampal neurons (7 DIV) stimulated with two concentrations of mouse TTR (55 and 300 *μ*g/ml), (two to three independent experiments, four to six neurons in each experiment). TTR administration was carried out at the '0 s' (line with dashes in the graph), but first time analyzed after stimulation is '0.33 s'. To address the role of megalin in TTR signaling activity, we used RAP, an LRP inhibitor and analyzed the phosphorylation levels of ERK (**g**, *n*=4–15), Akt (Ser473) (**h**, *n*=5–10) and CREB (Ser133) (I, *n*=3–12) following stimulation of TTR KO cultured hippocampal neurons (7 DIV) with recombinant mouse TTR (55 *μ*g/ml) in the presence or absence of RAP (350 *μ*g/ml, 30 min pre-incubation). The results are the average±S.E.M. of 3–15 independent experiments. TTR signaling activity was also addressed in megalin heterozygous TTR KO cultured hippocampal neurons (7 DIV) by analysis of the phosphorylation levels of ERK (**j**, *n*=3), Src (Tyr416) (**l**, *n*=4), Akt (Ser473) (**m**, *n*=3) and CREB (Ser133) (**n**, *n*=3) following stimulation with recombinant mouse TTR (55 *μ*g/ml) for 30 min. The results are the average±S.E.M. of three to four independent experiments. (**o**) Time course of normalized FRET/Donor values in cell body of YC-Nano15-transfected TTR KO and megalin (+/−) TTR KO cultured hippocampal neurons stimulated with mouse TTR (55 *μ*g/ml) (three independent experiments, five neurons in each experiment). (**p**) Same experimental design as in (**o**), but megalin (+/+) TTR KO littermate cultures were used and challenged with two concentrations of mouse TTR (55 and 300 *μ*g/ml) (one independent experiment for each, five neurons in each experiment). Statistical analysis was performed using one-way ANOVA followed by Bonferroni's multiple comparison test performed for each condition or Student's unpaired *t*-test for two-groups only comparisons. ***P<0.001, **P<0.01, *P<0.05, n.s., not significant as compared with the control or as indicated

We addressed the downstream signaling pathways triggered by WT TTR in the CNS at different levels: (i) phosphorylation of ERK and Akt (Ser473);^23^ (ii) intracellular calcium homeostasis;^23^ (iii) Src family kinase activation (Tyr416);^24^ (iv) gene transcription activation assessed by CREB phosphorylation (Ser133).^25^ Stimulation of TTR KO cultured hippocampal neurons with recombinant mouse TTR (55 *μ*g/ml), resulted in a significant rise of the phosphorylated levels of ERK (35%) and Akt (23%), 30 min after the stimulus (Figures 2a and c, respectively). ERK phosphorylation levels were higher in shorter time points (data not shown). TTR evoked a sustained (30–50%) activation of Src 30 min after stimulation (Figure 2b) and promoted a significant rise (20%) of CREB phosphorylation/activation (Figure 2d). As intracellular calcium was shown to induce neurite outgrowth,^26^ we performed FRET assays using a ultrasensitive calcium indicator-yellow Cameleon-Nano (YC-Nano15).^27^ TTR KO hippocampal neurons stimulated with mouse TTR showed a significant and transient rise in intracellular calcium concentration, in a dose-dependent manner (Figure 2f), compared with PBS (Supplementary Figure S1). This effect (calcium from extracellular source; Supplementary Figure S1) is observed mainly in neuronal cell body (where the results were obtained), was also seen in neurites (quantitative data not shown), demonstrated in the representative image of FRET assay (Figure 2e). To understand whether megalin mediates signaling cascades activated by TTR, we incubated TTR KO neurons with RAP, and observed that it blocked the phosphorylation of ERK (Figure 2g), Akt (Figure 2h) and CREB (Figure 2i). In the same manner, we stimulated megalin +/− TTR KO hippocampal neurons with TTR (55 *μ*g/ml), and observed that TTR stimulation was unable to activate ERK (Figure 2j), Src (Figure 2l), Akt (Figure 2m) and CREB (Figure 2n). The same happened with intracellular calcium, as detected by FRET (Figure 2o), in opposition to the megalin +/+ TTR KO littermate neurons (Figure 2p). Thus, these signaling pathways activated by TTR through megalin seem to be responsible for TTR neurite outgrowth.

### TTR transiently activates NMDA receptors through a megalin/Src mechanism

**Figure 3:**
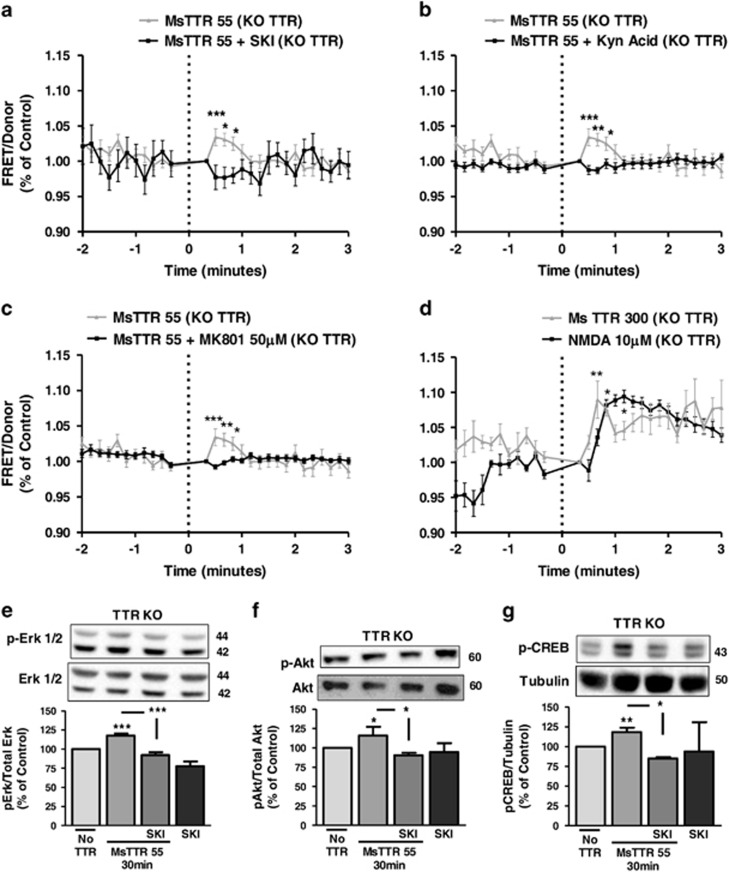
TTR transiently activates NMDA receptors through a megalin/Src mechanism. (**a**) Time course of normalized FRET/Donor values in cell body of YC-Nano15-transfected TTR KO cultured hippocampal neurons (7 DIV) stimulated with mouse TTR (55 *μ*g/ml) with or without Src inhibitor Ski (200 nM, 15 min pre-incubation) (for MsTTR, data are from Figure 2f and for MsTTR+SKI, three independent experiments, five to six neurons in each experiment). TTR administration was carried out at the '0 s' (line with dashes in the graph), but first time analyzed after stimulation is '0.33 s'. (**b, c** and **d**) Same experimental design as in (**a**) but using the NMDA/AMPA receptor inhibitor kynurenic acid (**b**, 2 mM, 15 min pre-incubation) (for MsTTR, data are from Figure 2f and for MsTTR+Kyn acid, three independent experiments, six neurons in each experiment), or the NMDA inhibitor MK801 (**c**, 50 *μ*M, 15 min pre-incubation) (for MsTTR, data are from Figure 2f and for MsTTR+MK801, three independent experiments, four to six neurons in each experiment) or stimulate neurons with mouse TTR (300 *μ*g/ml) *versus* an NMDA (10 *μ*M) stimulus (**d**, for MsTTR, data are from Figure 2f and for NMDA, one independent experiment, five neurons). To address the role of Src in TTR signaling activity, we used the Src inhibitor, SKI, and analyzed ERK (**e**, *n*=3–17), Akt (Ser473) (**f**, *n*=3–6) and CREB phosphorylation levels (Ser133) (**g**, *n*=3–6) following stimulation of TTR KO cultured hippocampal neurons (7 DIV) with recombinant mouse TTR (55 *μ*g/ml) with or without SKI (200 nM, 15 min pre-incubation). The results are the average±S.E.M. of 3–17 independent experiments. Statistical analysis was performed using one-way ANOVA followed by Bonferroni's multiple comparison test performed for each condition as compared with the control or as indicated. ***P<0.001, **P<0.01, *P<0.05, n.s., not significant

As megalin has a Src homology domain (SH3),^12^ and Src constitutes a crucial point of convergence for signaling pathways, we reasoned that Src could be the bridging molecule between megalin and downstream signaling pathways. We performed FRET assay with the calcium probe in TTR KO neurons incubated with TTR in the presence of SKI, a Src inhibitor, and concluded that SKI blocked intracellular calcium rise induced by TTR (Figure 3a). Then, we hypothesized that NMDA could be responsible for this increase in calcium, because Src regulates NMDA receptor activity.^28^ Both kynurenic acid (Figure 3b), a broader AMPA/NMDA inhibitor, and MK801 (Figure 3c), a specific NMDA inhibitor, blocked the increase in intracellular calcium promoted by TTR. In addition, 10 *μ*M NMDA stimuli promoted a similar increase in intracellular calcium as mouse TTR (300 *μ*g/ml) stimulus (Figure 3d). To understand whether Src was also governing TTR signaling pathways, we stimulated TTR KO cultured neurons with mouse TTR (55 *μ*g/ml) and SKI, and observed that it blocked the activation of ERK (Figure 3e), Akt (Figure 3f) and CREB (Figure 3g). Altogether, we concluded that Src is the key regulator of the signaling pathways triggered by TTR/megalin.

### TTR KO hippocampal neurons are more sensitive to an excitotoxic insult than WT counterparts

**Figure 4:**
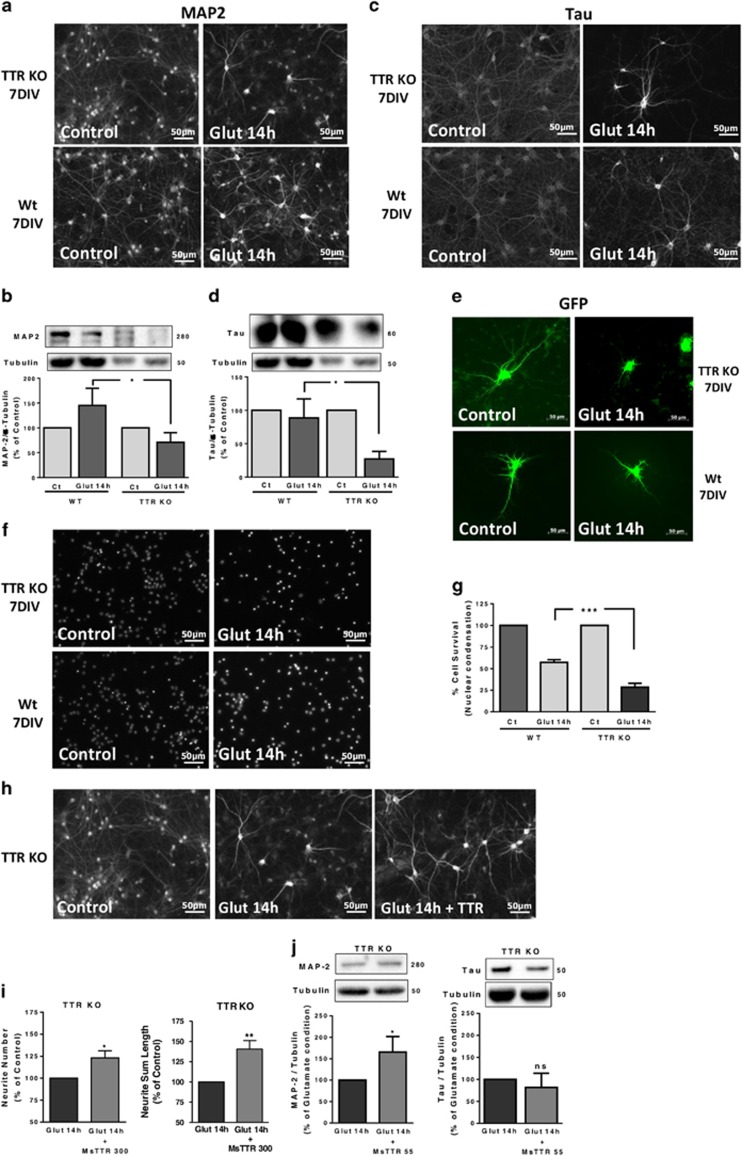
TTR KO hippocampal neurons are more sensitive to an excitotoxic insult, than WT counterparts. TTR KO and WT cultured hippocampal neurons (7 DIV) were subjected to excitotoxic stimulation with glutamate (125 *μ*M glutamate, 20 min) and further incubated in culture-conditioned medium (14 h). MAP_2_ (**b**, *n*=6) and Tau (**d**, *n*=5) protein levels were determined by western blot at the indicated time points after excitotoxic stimulation. The results are the average±S.E.M. of four to five independent experiments. Representative images of MAP_2_ (**a**) and Tau (**c**) are shown. GFP-transfected TTR KO and WT cultured hippocampal neurons (7 DIV) were also subjected to excitotoxic stimulation with glutamate. An immunocytochemistry against GFP was performed; the images are shown in (**e**), representative of four independent experiments. Cell survival was also assessed 14 h after the excitotoxic insult, using the fluorescent dye Hoechst 33342 (**f**, the representative image; **g**, WT *n*=3, TTR KO *n*=4). The results are the average±S.E.M. of three to four independent experiments. (**h** and **i**) TTR KO cultured hippocampal neurons (7 DIV) were subjected to excitotoxic stimulation with glutamate (125 *μ*M glutamate, 20 min) and further incubated in culture-conditioned medium with recombinant mouse TTR (300 *μ*g/ml) for 14 h, and stained for MAP_2_ (**h**). Neurite number and neurite sum length were quantified using ImageJ software (**i**). The results are the average±S.E.M. of three independent experiments. Using the same experimental design as in (**h**), MAP_2_ and Tau protein levels were also assessed by western blot, for the TTR KO cultured hippocampal neurons (**j**, *n*=4). Statistical analysis was performed using one-way ANOVA followed by Bonferroni's multiple comparison test performed for each condition or Student's unpaired *t*-test for two-groups only analysis. ***P<0.001, **P<0.01, *P<0.05, n.s., not significant as compared with the control or as indicated. All the scale bars presented correspond to 50 *μ*m

To address TTR neuroprotection, we subjected TTR KO and WT cultured hippocampal neurons to excitotoxic conditions, by a transient incubation with high glutamate concentration (125 *μ*M, for 20 min), and further incubating the neurons in culture-conditioned medium for 14 h. These conditions induce 40–50% apoptotic cell death,^29^ resembling neuronal death in penumbra of a stroke.^30^ TTR KO neurons are more sensitive to this excitotoxic insult than WT neurons, showing less viable dendrites in MAP_2_ immunocytochemistry assays (Figure 4a) and western blot data (Figure 4b). Axons, observed with Tau staining, are affected to the same extent as dendrites, with TTR KO neuronal cultures showing higher axonal degeneration than WT neuronal cultures (Figures 4c and d). The same was shown in green fluorescent protein (GFP)-transfected WT and TTR KO hippocampal neurons subjected to an excitotoxic insult 48 h after transfection, with transfected TTR KO neurons showing a much lower number of GFP-stained neurites than transfected WT neurons (Figure 4e). To assess the effect of TTR in cell death, we analyzed nuclear morphology by Hoechst 33342, 14 h after the excitotoxic insult. TTR KO neuronal cultures showed significantly more dead neurons than WT counterparts (Figures 4f and g). TTR could prevent neurite degeneration induced by glutamate stimulation, when added (300 *μ*g/ml) after the excitotoxic insult, observed by MAP_2_ immunostaining (Figure 4h), neurite quantification (Figure 4i) and MAP_2_ western blot (Figure 4j). Tau protein levels were not rescued (Figure 4j), indicating that TTR is mostly neuroprotective at dendrite level.

### TTR promotes neuronal survival, through megalin

**Figure 5:**
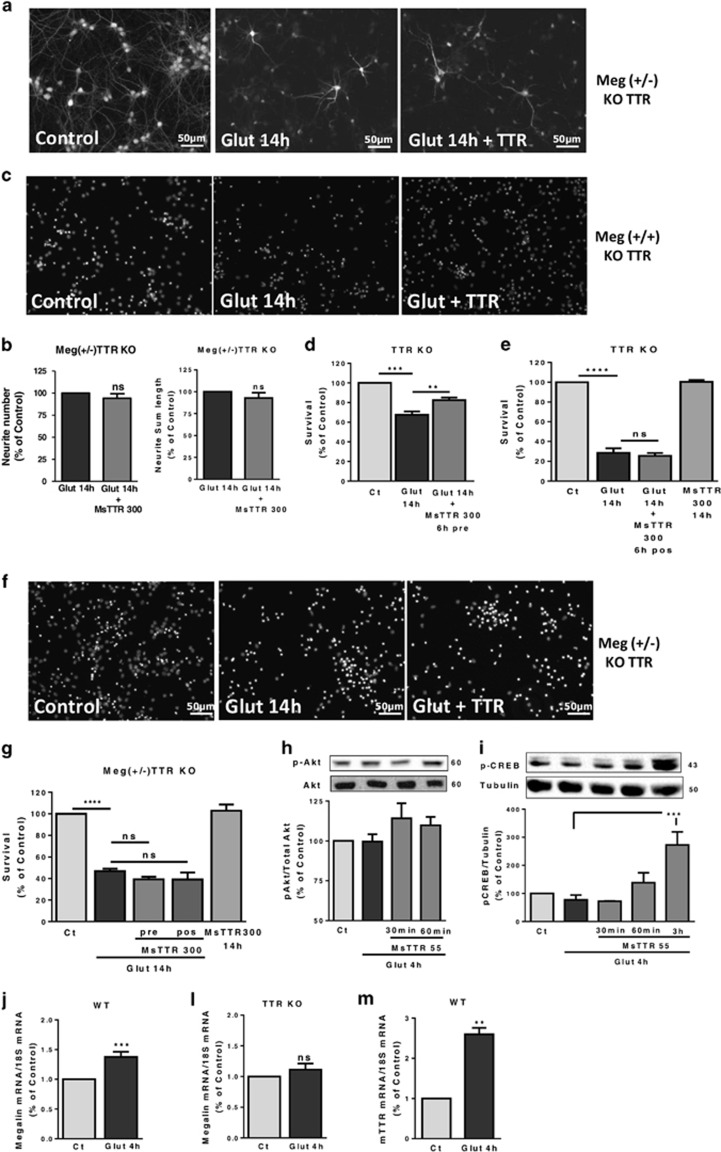
TTR promotes neuronal survival, through a megalin-dependent manner. (**a**) Megalin (+/−) TTR KO cultured hippocampal neurons (7 DIV) were subjected to excitotoxic stimulation with glutamate (125 *μ*M glutamate, 20 min) and further incubated in culture-conditioned medium with recombinant mouse TTR (300 *μ*g/ml) for 14 h, and stained for MAP_2_. Representative images are shown (**a**) and neurite number and neurite sum length were quantified with ImageJ software (**b**). The results are the average±S.E.M. of nine independent experiments. (**c**) TTR KO cultured hippocampal neurons (7 DIV) were subjected to excitotoxic stimulation with glutamate (125 *μ*M glutamate, 20 min), with a 6 h pre-incubation with recombinant mouse TTR (300 *μ*g/ml) in culture-conditioned medium. Cell survival was accessed 14 h after the excitotoxic insult, using the fluorescent dye Hoechst 33342 (**c**, representative images; **d**). The results are the average±S.E.M. of five independent experiments. In (**e**), a similar experimental design as in (**b**) was used, but adding mouse TTR (300 *μ*g/ml) after the excitotoxic stimulus. The results are the average±S.E.M. of four independent experiments. (**g**) Using the same approach used as in (**d**) and (**e**), but using megalin (+/−)TTR KO cultured hippocampal neurons (7 DIV), mouse TTR (300 *μ*g/ml) was added before and after the excitotoxic stimulus. The results are the average±S.E.M. of five independent experiments (**f**). To address TTR signaling activity after the excitotoxic stimulation, mouse TTR was added to TTR KO hippocampal cultures (7 DIV), 4 h after a glutamate stimulus (125 *μ*M glutamate, 20 min) and Akt (Ser473) (**h**, *n*=3) and CREB phosphorylation (Ser133) (**i**, *n*=3–5) were analyzed by western blot at several time points (30 min, 60 min, 3 h). Megalin mRNA levels were also accessed after an excitotoxic insult (125 *μ*M glutamate, 20 min) in WT cultured hippocampal neurons (7 DIV) (**j**, *n*=5) and in TTR KO cultured hippocampal neurons (7 DIV) (**l**, *n*=9). TTR mRNA levels were also analyzed after an excitotoxic insult (125 *μ*M glutamate, 20 min) in WT cultured hippocampal neurons (7 DIV) (**m**, *n*=4). The results are the average±S.E.M. of four to five independent experiments. Statistical analysis was performed using one-way ANOVA followed by Bonferroni's multiple comparison test performed for each condition as compared with the control or as indicated or Student's unpaired *t*-test for two-groups only analysis. ***P<0.001, **P<0.01, *P<0.05, n.s., not significant. All the scale bars presented correspond to 50 *μ*m

To investigate the interplay between TTR and megalin in CNS neuroprotection, we subjected megalin (+/−) TTR KO hippocampal neurons to an excitotoxic insult, and added TTR after the insult. We observed that TTR did not prevent dendrite degeneration induced by glutamate, both in dendrite number and total length (Figures 5a and b). To understand whether TTR also rescues neuronal survival with/without megalin, we pre-incubated TTR KO neurons with TTR (300 *μ*g/ml), 6 h before excitotoxic stimulus, and observed a reduced cell death (Figures 5c and d). When added after glutamate, TTR was unable to protect neurons (Figure 5e). To understand whether megalin was involved in TTR neuroprotection at neuronal survival level, we performed the same experimental design using megalin (+/−) TTR KO neurons. We realized that neither TTR pre- or post-incubation, 300 or 55 *μ*g/ml (data not shown), could protect to any extent cell survival (Figures 5f and g). Megalin (+/+) TTR KO littermates show the same level of neuroprotection observed in TTR KO cultures (data not shown). We found that TTR was capable of activating phospho-CREB (Figure 5i), and probably Akt, with a tendency shown (Figure 5h), in excitotoxic conditions. As megalin was shown to be necessary for the neuroprotective action of TTR, we quantified megalin mRNA levels, after glutamate stimulation (4 h), in both TTR KO and WT cultures. Megalin mRNA was significantly upregulated in WT cultures (Figure 5j), in opposition to TTR KO cultures (Figure 5i). In WT neurons, there is the possibility of endogenous TTR being produced (although not consensual).^31^ We did not detect TTR protein by western blot neither in cell extracts nor in culture-conditioned medium (data not shown). However, we found that TTR mRNA is highly upregulated in these cultures after excitotoxic stimulus (4 h) (Figure 5m).

### TTR neuroprotection involves the activation of anti-apoptotic signaling pathways

**Figure 6:**
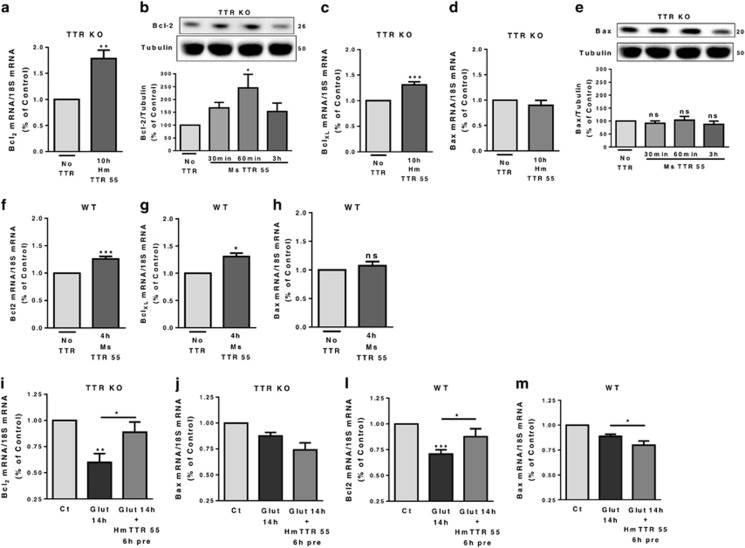
TTR neuroprotection involves the activation of anti-apoptotic signaling pathways. TTR KO cultured hippocampal neurons (7 DIV) were stimulated with recombinant mouse/human TTR (55 *μ*g/ml) in culture-conditioned medium. Anti-apoptotic proteins levels were accessed. Bcl2 mRNA levels (**a**, *n*=5) and protein levels (**b**, *n*=6-8) and BclXL mRNA levels (**c**, *n*=4). The results are the average±S.E.M. of four to eight independent experiments. Pro-apoptotic levels of Bax were also accessed by analysis of mRNA (**d**, *n*=4) and protein levels (**e**, *n*=5–7). The results are the average±S.E.M. of four to seven in independent experiments. WT cultured hippocampal neurons (7 DIV) were also stimulated with recombinant mouse TTR (55 *μ*g/ml) and the mRNA of Bcl2 protein family members were accessed: Bcl2 (**f**, *n*=4), BclXL (**g**, *n*=3) and Bax (**h**, *n*=4). To address TTR neuroprotective activity through the balance of anti-apoptotic signaling pathways, Bcl2 and Bax mRNA levels were quantified in excitotoxic conditions with/without human TTR pre-incubation (55 *μ*g/ml) in TTR KO cultured hippocampal neurons (7 DIV) (**i**, *n*=4; **j**, *n*=3) and WT cultured hippocampal neurons (7 DIV) (**l**, *n*=3–5; **m**, *n*=3–5). The results are the average±S.E.M. of three to six independent experiments. Statistical analysis was performed using one-way ANOVA followed by Bonferroni's multiple comparison test performed for each condition or Student's unpaired *t*-test for two-group comparisons. ***P<0.001, **P<0.01, *P<0.05, n.s., not significant as compared with the control or as indicated

As CREB was activated when TTR was added, after excitotoxic conditions (Figure 5i), we investigated whether phospho-CREB activates anti-apoptotic genes, such as members of Bcl2 protein family.^32^ We stimulated TTR KO neurons with TTR (human, 55 *μ*g/ml) and observed that Bcl2 (Figure 6a) and BclXL mRNA levels (Figure 6c) were highly upregulated. Bcl2 protein levels were also upregulated 60 min upon TTR stimulation (Figure 6b). Bax was not altered at the mRNA level (Figure 6d) or protein level (Figure 6e). In WT neurons, a similar pattern was seen, that is, upregulation of Bcl2 (Figure 6f) and BclXL mRNA levels (Figure 6g), and no change in Bax mRNA (Figure 6h). We expected the balance Bcl2/Bax levels governing cell death to be more relevant in excitotoxic conditions, for cell survival.^33^ Bcl2 mRNA levels were downregulated after glutamate stimulation in both WT (Figure 6l) and TTR KO cultures (Figure 6i). TTR pre-incubation before excitotoxic stimulus resulted in rescue of Bcl2 downregulation, in both TTR KO (Figure 6i) and WT cultures (Figure 6l). Regarding Bax mRNA levels, they did not change significantly after glutamate stimulation in both WT (Figure 6m) and TTR KO cultures (Figure 6j). Yet, when TTR was pre-incubated before the excitotoxic stimulus, Bax levels were significantly downregulated in the WT neuronal cultures (Figure 6m), and with a clear tendency for downregulation in TTR KO cultures (Figure 6j). Taken together, the results clearly indicate that TTR shifts the balance of the Bcl2 protein family members toward anti-apoptotic proteins resulting in less neuronal death.

### TTR neuroprotection *in vivo* (pMCAO) is also megalin-dependent

**Figure 7:**
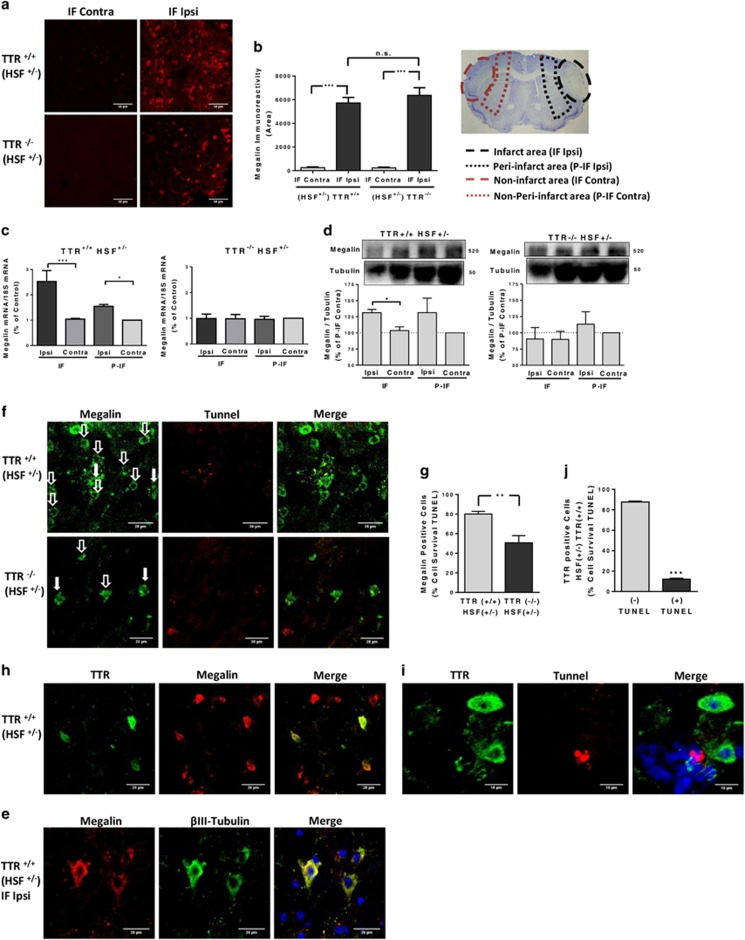
TTR neuroprotection *in vivo* (pMCAO) is also megalin-dependent. (**a**) Megalin levels after pMCAO were accessed by immunofluorescence confocal stacks of the different brain areas of wild-type and TTR KO mice (HSF+/− background) after 24 h of pMCAO. Representative immunofluorescence confocal stack of megalin in infarct area (IF Ipsi) and contralateral area (IF Contra) (scale bar 50 *μ*m). (**b**) The semi-quantification results are the average±S.E.M. of three to five stacks/animal (three animals for each phenotype). (**c**) Megalin mRNA levels were quantified for the different brain areas (IF Contra *versus* IF Ipsi; P-IF Contra *versus* P-IF Ipsi) of the 24 h pMCAO WT and TTR KO mice (HSF+/− background). The results are the average±S.E.M. of three to four different animals for each phenotype. (**d**) Megalin protein levels were quantified for the different brain areas (IF Contra *versus* IF Ipsi) of the 24 h pMCAO WT and TTR KO mice (HSF+/− background). The results are the average±S.E.M. of three different animals for each phenotype. (**e**) Representative images of megalin immunofluorescence confocal stacks of live cells co-localizing with *β*III-tubulin from the infarct area of WT mice (HSF+/− background) after 24 h pMCAO (three animals). (**f** and **g**) Semi-quantification of the cell survival accessed with TUNEL reaction assay from the megalin-positive cells in the infarct area of TTR KO and WT mice (HSF+/− background) after 24 h pMCAO. The results are the average±S.E.M. of five to six stacks from three animals for each phenotype (**f**, representative image; **g**). (**h**) Representative immunofluorescence confocal stack of TTR-positive live cells co-localizing with megalin, from the infarct area of WT mice (HSF+/− background), after 24 h pMCAO (three animals). (**i** and **j**) Semi-quantification of the cell survival from the TTR-positive cells in the infarct area of WT mice (HSF+/− background) after 24 h pMCAO. Cell survival was accessed with TUNEL reaction assay. The results are the average±S.E.M. of six stacks from three WT pMCAO animals (**i**, representative images; **j**). Statistical analysis was performed using one-way ANOVA followed by Bonferroni's multiple comparison test performed for each condition or Student's unpaired *t*-test for two-group comparisons. ***P<0.001, **P<0.01, *P<0.05, n.s., not significant as compared with the control or as indicated. Scale bar in (**a** and **i**) correspond to 50 and 10 *μ*m, respectively. All the others (**e,f**and **h**) correspond to 20 *μ*m

Having identified megalin as the receptor involved in the transduction of TTR neuroprotection in hippocampal neuronal cell culture, we investigated whether megalin was involved and/or affected in a mice stroke model– pMCAO. Wild-type TTR and TTR KO mice (both HSF+/− background) were subjected to ischemia by pMCAO and analyzed 24 h later. Megalin and TTR (only in WT TTR mice, data not shown) immunoreactivity was increased, in the infarct area only, of both WT TTR and TTR KO mice (Figures 7a and b). To clarify megalin upregulation *in vivo*, we quantified megalin mRNA and protein, in different brain areas of TTR KO and WT pMCAO mice. We found that WT mice upregulated megalin protein and mRNA in the infarct areas (IF Ipsi;P-IF Ipsi) *versus* corresponding non-infarct areas (IF-Contra;P-IF Contra) (Figures 7c and d). In opposition, megalin protein and mRNA in TTR KO mice are unchanged between the corresponding brain areas (Figures 7c and d). RAGE and IGF-IR (also bind TTR) did not show any changes between WT and TTR KO mice (data not shown). Through immunohistochemistry, we found that cells that were upregulating megalin in the infarct area of the pMCAO WT mice were neurons, as they fully co-localize with *β*III-tubulin (Figure 7e). Moreover, we unraveled, through TUNEL staining, that most of megalin-positive cells in WT TTR mice were alive (80%), whereas in KO TTR mice, only 50% survived (Figures 7f and g). Santos *et al.*^3^ also demonstrated that TTR-positive cells were neurons, as we confirmed in this work, but did not clarify whether neurons were alive or dead. Via immunohistochemistry, we observed that all the TTR-positive cells co-localize with megalin (Figure 7h). In addition, by triple staining TTR, TUNEL and Hoechst 33342, we found that most of TTR-positive neurons in WT TTR mice were alive (90%) (Figures 7i and j).

## Discussion

In this study, we show that TTR has megalin-dependent neuritogenic activity on cultured hippocampal neurons, either from WT or TTR KO mice under physiologic conditions. More meaningful is the fact that TTR neuronal action is important in pathological/excitotoxic conditions. TTR is neuroprotective both for neuronal survival and neurite preservation under excitotoxic conditions, through the activation of well-known neuroprotective megalin-dependent signaling pathways and transcription factors. In addition, we demonstrate that TTR neuroprotective role in a focal cerebral ischemia model is also megalin-dependent.

The neuritogenic activity that we describe for TTR in CNS neurons (Figures 1a and b) has been observed previously in the PNS (dorsal root ganglia neurons),^2, 34^ indicating a widespread action over neuronal populations. Moreover, we describe that TTR function in promoting neurite outgrowth is independent of its ligands, a similar effect observed by Fleming *et al.*^34^ in the PNS. Other studies described that TTR KO mice have spatial learning and memory deficits, in Morris water maze testing.^35, 36^ TTR has also been associated with the maintenance of memory capacities during aging, because its hippocampal gene expression is downregulated in aged animals.^37^ So, this neuritogenic activity of TTR might be correlated with this role of TTR in memory and learning, as seen for other molecules.^38^ Although it can be due to other TTR pathways, a connection to be explored in the future. In agreement, TTR was found to be highly upregulated in the hippocampus of mice under an enriched environment housing compared with standard housing controls,^39^ indicating that it might be important to sustain enhanced memory function and recovery from lesion (enriched environment *versus* standard housing).

Megalin was shown to be important in the neuritogenic activity of other molecules such as metallothioneins^40^ and *α*2-macroglobulin (*α*2-M).^25^ We now demonstrate that TTR neuritogenic activity in cultured hippocampal neurons (CNS) is mediated by megalin, (Figures 2a and d), whereas LRP1 does not seem to be involved.

We found that TTR interaction with megalin activates the MAP kinase, ERK, Akt and Src, which ultimately lead to the upregulation of the CREB transcription factor (Figures 3a and d), as seen for other LRP ligands. TTR also contributes to a significant rise in intracellular calcium (Figure 3g) that might underlie the neurite outgrowth triggered by TTR. Similarly, *α*2-M was shown to promote neurite outgrowth in cortical neurons through ERK1/2, CREB and intracellular calcium changes,^25^ but in this case, LRP1 was involved.^24^ We show that the rise in intracellular calcium promoted by TTR occurs through NMDA receptors regulated by Src (Figure 5), allowing more calcium influx to neurons, probably through the phosphorylation of Nr2A/2B subunits, increasing channel gating and the probability of the channel to be in the open state.^28^ A similar activation of NMDA receptors by other LRP receptors was described by Qiu *et al.*^41^ for *α*2-M. Moreover, the Src kinase family has been described as a crucial point of convergence for signaling pathways that subsequently enhance NMDA receptor activity.^28^ This can also be a neuroprotective strategy, because it has been already demonstrated in a global ischemia model that NR2A receptor activates ERK1/2-CREB signaling pathways, that ultimately enhance BDNF and Bcl2 expression, decreasing hippocampal CA1 neuronal death.^42^

Importantly, we demonstrated that TTR KO neurons are more sensitive to excitotoxic insults than WT counterparts, both at neuronal survival and neurite degeneration levels, and that TTR could partially rescue dendrites if added after the insult (Figure 6). This neurite neuroprotection will allow a higher trophic support by the neurotrophic factors (such as BDNF, NGF and TTR) that are exchanged between neuronal processes, allowing the neuronal network to survive and be more resistant to deleterious stimuli.^43, 44^ In a similar manner, TTR was shown to be important in nerve regeneration, as TTR KO mice have dorsal root ganglia neuron neurite outgrowth impairment and decreased regeneration after never injury.^34^ Moreover, local delivery of TTR to the crushed nerves rescues the regeneration phenotype of TTR KO mice.^2^ Regarding cell survival, TTR was only able to partially protect neurons when added before, but not after, the excitotoxic insult (Figures 7b and c), indicating that the neuroprotective role of TTR in cell survival probably requires *de novo* protein synthesis. Altogether, neuroprotection by TTR acts in excitotoxic conditions of neuronal cultures and in pMCAO model in a megalin-dependent manner, involving the signaling pathways described above. A recent work also points for megalin as a preponderant receptor for cell proliferation and survival, as expression of megalin was observed in the majority of malignant melanoma tumors (60%) *versus* benign counterparts (20%).^45^

TTR shifts the balance of Bcl2 proteins toward anti-apoptotic proteins resulting in reduced cell death. These results resemble those observed in a kidney cell line, where NGAL (neutrophil gelatinase-associated lipocain) was able to reduce apoptosis in a hypoxia-reperfusion model, by regulating Bax and Bcl2 levels, in a megalin-dependent manner.^46^ The control of Bcl2 proteins has also been suggested as a strategy to treat neurodegenerative disorders, such as Parkinson.^33^

In a murine model of Alzheimer disease, it was described that neurons transcribe TTR mRNA and secrete the protein in small amounts, contributing to a neuroprotective effect against A*β* aggregates.^31^ We verified that TTR mRNA levels were also upregulated in ischemic challenged WT neuronal cultures (Figure 7m). TTR was also shown to be upregulated (in plasma) in ischemic preconditioning both in rats^47^ and humans.^48^ In a transient focal cerebral ischemia model, TTR was upregulated in CSF.^49^

We found in pMCAO mice that neurons that overexpress megalin in the infarct areas of WT mice survive significantly more than the neurons of TTR KO mice, confirming the role of megalin in neuroprotection induced by TTR *in vivo* (Figures 7f and g). Besides, we found that most of the neurons in the infarct area of WT mice also upregulate TTR and megalin (Figure 7h), and almost all are alive (Figures 7j and i), like we observed for WT neuronal cultures after an excitotoxic insult (Figure 4 f). These results definitely indicate that megalin is responsible for the TTR-induced neuroprotection in cerebral ischemia. Hence, the upregulation of TTR in CSF and/or megalin neuronal upregulation should be addressed as potential preventive therapies for stroke.

Together, these findings indicate that TTR could be described as a new neurotrophic factor, which promotes neurite outgrowth in physiological conditions through upregulation of intracellular calcium and a Src/ErK/Akt/CREB pathway in a megalin-dependent manner, and is neuroprotective at different levels in pathological ischemic conditions also through megalin, either *in vitro* and *in vivo* as depicted in Supplementary Figure S2. These two interrelated proteins, megalin and TTR, could be further explored as possible endogenous neuroprotective targets, which can be potentiated in a novel preventive/prognostic strategy in different pathologies, certainly in stroke.

## Materials and Methods

### Animals

The number of mice handled for this research was approved by the Institutional and National General Veterinary Board Ethical Committees according to National and European Union rules. Three- to six-month-old TTR wild-type (+/+), TTR KO (−/−) ^50^ and megalin (+/−) TTR KO mice in a 129/svJ background were used for the hippocampal neuronal cultures. Three to six-month-old TTR KO (−/−) and control littermates under a HSF1 (+/−) background (129/svJ) were used for the pMCAO experiments.^3^ Megalin heterozygous mice were kindly provided by Dr. Thomas Willnow, Max-Delbrueck Center for Molecular Medicine, Berlin, Germany.^51^ The animals were maintained under a 12 h light/dark cycle in type II cages in specific pathogen-free conditions (microbiological heath status available). Animals were fed with regular rodents chow and tap water *ad libitum*. Genotypes were determined from tail-extracted genomic DNA, using primers for the detection of exon 2 of TTR (which is disrupted in TTR−/− by insertion of a neomycin resistance gene), megalin and neomycin as previously described.^50, 51^ ARRIVE guidelines for reporting animal research were taken into consideration in the reporting of the experiments.

### Recombinant TTR production and purification

Recombinant mouse and human TTR were produced in a bacterial expression system using *Escherichia coli* BL21 and purified as previously described.^52^ Briefly, after growing the bacteria, the protein was isolated and purified by preparative gel electrophoresis after ion-exchange chromatography. Protein concentration was determined using the Lowry method.

### Recombinant GST-RAP production and purification

Recombinant GST-RAP production was previously described.^22^ Briefly, expression of the GST-RAP (pGEX-RAP) was induced by treating an *Escherichia coli* BL21 culture in the exponential phase of growth (A600 nm= 0.4–0.5) with 0.5 mM isopropyl *β*-D-thiogalactoside for 4–6 h at 30 °C. The protein, with an apparent molecular mass of 65KDa (GST (25 KDa)+ RAP (39KDa)) in a SDS-PAGE gel, was extracted and purified from the bacterial pellet through affinity chromatography on glutathione Sepharose 4B (GE Healthcare, Little Chalfont, UK), according to the manufacturer's recommendations.

### Endotoxin removal

To remove endotoxins from recombinant TTR and GST-RAP, a polymixin B column (Thermo Scientific, Waltham, MA, USA) was used. Briefly, the column was regenerated with 1% sodium deoxycholate (Sigma, St Louis, MO, USA) and washed with pyrogen-free buffer to remove detergent. Recombinant TTR was applied to the column and incubated during 1 h at room temperature. Aliquots of pyrogen-free buffer were added and the flow-through was collected. Protein concentration was determined using the Bradford method.

### Primary hippocampal neuronal cultures

Primary cultures of mouse hippocampal neurons were prepared from the hippocampus of E17-E18 of WT, TTR KO and megalin (+/−)/(+/+) TTR KO mice embryos (129/svJ background), as previously described.^29, 53^ Neuronal cultures were maintained in serum-free Neurobasal medium (Gibco, Life Technologies, Carlsbad, CA, USA), supplemented with B27 (Gibco, Life Technologies), glutamate (25 *μ*M), glutamine (0.5 mM) and gentamicin (0.12 mg/ml). Cells were kept at 37 °C in a humidified incubator with 5% CO2/95% air, for 24 h, for the neurite outgrowth experiments or 7 days for the western blot experiments, the time required for maturation of hippocampal neurons. Cells were cultured at a density of 90 000 cells/cm^2^ or 80 000 cells/cm^2^ on poly-D-lysine-coated 6-well microplates (MW6) (for western blot and real-time PCR experiments) or glass coverslips (for immunocytochemistry studies), respectively. Megalin (+/−) TTR KO mice embryos were all genotyped (5 h express protocol) to separate the megalin (+/−) from the (+/+). Meanwhile, hippocampi were hibernated using Hibernate E medium (Gibco, Life Technologies, Carlsbad, CA, USA) supplemented with diluted 1:10 B27 (Gibco, Life Technologies) and kept at 4 ºC. The hippocampal neurons were cultured using a serum-free medium (B27), which does not contain T4 or RBP – TTR ligands. The following pre-established exclusion criteria were used: cell cultures with high % of cell death (> 30%) and/or neurite network undeveloped or damaged. For the SKI-1 (Src kinase inhibitor I, Abcam (Cambridge, UK), ab120839, 200 nM) experiments, cells were pre-incubated with the inhibitor for 15 min and stimulated with TTR (55 *μ*g/ml) in the presence or absence of the drug, for the time mentioned, at 37 ºC. Regarding RAP (receptor-associated protein; LRP inhibitor, custom expressed, 350 *μ*g/ml) experiments, cells were pre-incubated with the inhibitor for 30 min and then stimulated with TTR (55 *μ*g/ml) in the presence or absence of the protein, for the time mentioned, at 37 ºC.

### Neurite outgrowth analysis

Cultured hippocampal neurons from WT, TTR KO or megalin (+/−) TTR KO mice embryos were isolated under the above-described conditions, plated on poly-D-lysine-coated glass coverslips at a density of 5 × 10^4^ cells/cm^2^. Recombinant mouse/human TTR (55 *μ*g/ml and 300 *μ*g/ml) was added to cell culture medium immediately after plating. Cells were maintained in culture during 24 h, approximately, in order to allow the precise tracing of all the neurites per neuron. Longer days *in vitro* do not allow precise neurite quantification per neuron. Neurite outgrowth assay was also performed in 7 DIV TTR KO and megalin (+/−) TTR KO cultures, after an excitotoxic stimulus, which allowed the neurite quantification. Cells were fixed with 4% paraformaldehyde and immunofluorescence was performed against MAP_2_ (1:800, Abcam). The coverslips were mounted in a fluorescent mounting medium (DAKO, Glostrup, Denmark) and imaging was performed on a Zeiss (Oberkochen, Germany) AxioImager Z1microscope, using a × 20 oil objective. Between 15 and 20 pictures were randomly taken throughout the coverslip, in a blinded manner, for each condition. For the experiments involving the TTR antibody, TTR was pre-incubated 1 h at 37 °C with the antibody, before being added to the cultures. Regarding RAP experiments, RAP (350 *μ*g/ml) was added 30 min before TTR was added to the cultured neurons, after plating. Morphological measurements of neurite outgrowth were performed using the plugin NeuronJ from the ImageJ software.^54^ Number and sum length of neurites per cell were the analyzed parameters. At least 60–80 cells were counted for each experimental condition in a blinded manner, and the experiments were repeated in more than three independent preparations. The experimental unit in these assays was each individual culture (with more than 60 neurons analyzed in each individual culture).

### Cell death assay

Hippocampal neurons were cultured for 7 days on poly-D-lysine-coated glass coverslips as previously described. After the experiments, cells were fixed in 4% sucrose/4% paraformaldehyde (in PBS). The cells were washed twice with PBS and incubated with Hoechst 33342 (0.5 *μ*g/ml) to stain nuclei. Analysis of the nuclear morphology was performed on Zeiss AxioImager Z1 florescence microscope, under a × 40 oil objective. Live and dead cells were counted in a blinded manner, as performed for neurite outgrowth analysis, using ImageJ. The experimental unit in these assays was each individual culture (always performed with different breeding females in independent neuronal culture isolation procedures).

### Western blot analysis

Cultured hippocampal neurons and dissected brain areas from pMCAO mice were homogenized in lysis buffer containing 20 mM MOPS, 2 mM EGTA, 5 mM EDTA, 30 mM sodium fluoride, 60 mM glycerophosphate, 20 mM sodium pyrophosphate, 1 mM sodium orthovanadate, 1 mM phenylmethylsulphonyl fluoride, 1% Triton X-100 and 1 × protease inhibitors mixture (GE Healthcare). Total protein concentration was determined using the Bradford method. Fifty micrograms of protein were applied and separated by 4%/10% Tris-Glycine SDS-PAGE (or 3%/7% Tris-Acetate polyacrylamide gels for megalin) and transferred to a nitrocellulose Hybond-C membrane (GE Healthcare), using a wet system, with Tris/Glycine/SDS buffer (Bio-Rad, Hercules, CA, USA). Membranes were blocked at least 1 h at room temperature in blocking buffer, 5% bovine serum albumin in phosphate-buffered saline Tween 20 (PBST), and then incubated overnight a 4°C with primary antibodies diluted in blocking buffer, namely sheep megalin (1:1000; custom made), rabbit megalin (1:750, Abcam, ab129198), rabbit LRP1 (1:5000, Abcam, ab92544), rabbit Bax (1:1000, Cell Signaling (Danvers, MA, USA), #2772), rabbit Bcl2 (1:1000, Cell Signaling, #2870), rabbit phospho-Akt (Ser473, 1:1000, Cell Signaling, #4060), rabbit Akt (1:1000, Cell Signaling, #9272), rabbit phospho-p44/42 MAPK (1:1000, Cell Signaling, #9101), rabbit p44/42 MAPK (1:1000, Cell Signaling, #9102), rabbit phospho-CREB (Ser133, 1:1000, Cell Signaling, #9198), rabbit CREB (1:1000, Cell Signaling, #9197), rabbit phospho-Src (Tyr416, 1:1000, Cell Signaling, #6943), mouse Src (1:1000, Cell Signaling, #2110), rabbit anti-MAP2 (1:800; Abcam, ab24640), mouse anti-Tau (1:750, Cell Signaling, #4019), mouse *α*-tubulin (1:10 000, Sigma, T8203). Membranes were then incubated with anti-rabbit IgG-HRP (1:10 000, Binding Site, Birmingham, UK) and anti-mouse IgG-HPR (1:5000, Binding Site), for 1 h at room temperature. Blots were developed using Immun-Star WesternC Chemiluminescent kit (Bio-Rad) and exposed to Bio-Rad ChemiDoc XRS system or ECL Hyperfilm (GE Healthcare), if signal was too low. Quantitative analyses were performed using the Quantity One software or ImageLab from Bio-Rad Laboratories. The experimental unit in western blot assays was each individual culture (always performed with different breeding females in independent neuronal culture isolation procedures).

### mRNA semi-quantification through real-time PCR

Total RNA was extracted from either 7 DIV cultured hippocampal neurons or dissected brain areas from pMCAO mice using TRIzol Reagent (Thermo Fisher Scientific, Waltham, MA, USA), as previously described.^55^ RNA quality and integrity was assessed using the Experion automated gel electrophoresis system (Bio-Rad, Hercules, CA, USA), as previously described.^55^ Samples showing RNA degradation or contamination by DNA were discarded. RNA concentration was determined using NanoDrop 1000 (Thermo Scientific). The samples were aliquoted and stored at −80 °C until further use. cDNA synthesis was performed using 1 *μ*g of total RNA and the SuperScript cDNA synthesis (Life Technologies, Carlsbad, CA, USA), as previously described.^55^ Samples were stored at −80 °C until further use. Primers used for real-time PCR were designed using 'Beacon Designer' software (Premier Biosoft International, Palo Alto, CA, USA) as described previously.^55^ Oligonucleotides used for megalin real-time PCR were: forward 5′-GGCTCACTCAAGTCCGCATCTTCC-3′ and reverse 5′-ACTCAACGGTGCTGCCAGTTACG-3′ for Bcl2: forward 5′-TGTGGATGACTGAGTACCT-3′ and reverse 5′-CAGAGACAGCCAGGAGAA-3′ for BclXL: forward 5′-GCCACCTATCTGAATGACCA-3′ and reverse 5′-GTTCCCGTAGAGATCCACAAA-3′ for Bax: forward 5′-TAAAGTGCCCGAGCTGAT-3′ and reverse 5′-CCGAAGTAGGAGAGGAGG-3′ for mTTR: forward 5′-AGCCCTTTGCCTCTGGGAAGA-3′ and reverse 5′-TGCGATGGTGTAGTGGCGATGG-3′. 18S RNA was used as reference gene with the following primers: forward 5′-AAATCAGTTATGGTTCCTTTGGTC-3′ and reverse 5′GCTCTAGAATTACCACAGTTATCCAA3′. The annealing temperature was 60 °C. For gene expression analysis, 1 *μ*l of 1:10 diluted cDNA was added to 10 *μ*l of 2 × SYBR Green Master Mix (Bio-Rad) and the final concentration of each primer was 250 nM in 20 *μ*l total volume. The thermocycling reaction was initiated by activation of Taq DNA Polymerase by heating at 95 °C during 3 min, followed by 45 cycles of a 15 s denaturation step at 95 °C and a 20 s annealing/elongation step at 60 °C. The fluorescence was measured after the extension step, using the iQ5 Multicolor Real-Time PCR Detection System (Bio-Rad). After the thermocycling reaction, the melting step was performed with slow heating, starting at 55 °C and with a rate of 0.5 °C per 10 s, up to 95 °C, with continuous measurement of fluorescence. Data analysis was performed using Pfaff method that adds the efficiency correction (amplification efficiencies of transcripts) to the ΔCP (between target gene and reference gene).^56^ Results were normalized with 18S RNA as internal reference gene, because it showed a stable expression in the conditions tested (compared with other reference genes tested). Efficiency correction was carried out for all the independent experiments, in all PCR plates. The experimental unit in qPCR was each individual culture (always performed with different breeding females in independent neuronal culture isolation procedures).

### Transfection

The expression vector containing the GFP gene was the pEGFP-N1 vector (BD Biosciences Clontech, San Jose, CA, USA). The plasmid sequence of pEGFP-N1 was verified by DNA sequencing reactions. Transfection of cultured hippocampal neurons with GFP and yellow cameleon-Nano15 was performed by the calcium phosphate co-precipitation method as previously described, with minor modifications.^53^ Briefly, 2 *μ*g of plasmid DNA were diluted in Tris-EDTA (TE) pH 7.3 and mixed with HEPES calcium chloride pH 7.2 (2.5 M CaCl2, 10 mM HEPES). This DNA/TE/Calcium mix was added to an 2 × HEPES-buffered saline solution (270 mM NaCl, 10 mM KCl, 1.4 mM Na2HPO4, 11 mM Dextrose, 42 mM HEPES), pH 7.2. The precipitates were allowed to form for 30 min, with vortex mixing every 5 min, to ensure that the precipitates had similar small sizes. Meanwhile, coverslips with cultured neurons were incubated with cultured conditioned medium with 2 mM of kynurenic acid. The precipitate was added drop wise to each coverslip and incubated at 37 °C, 5% CO_2_, for 3 h. Cells were then washed with acidic (10% CO_2_) equilibrated culture medium containing 2 mM kynurenic acid and returned to the 37 °C/5% CO_2_ incubator for 15 min. Finally, the medium was replaced with the initial culture-conditioned medium, and the cells were further incubated in a 37 °C/5% CO_2_ incubator for 48 h to allow protein expression.

### Immunocytochemistry

Cells were fixed in 4% sucrose/paraformaldehyde and permeabilized with 0.3% Triton X-100 in PBS. Neurons were then incubated with 5% bovine serum albumin (Sigma) in PBS+0.1% Tween 20, for 1 h at 37 °C, to block nonspecific binding, and incubated with primary antibodies, overnight at 4 °C. Cells were then washed five times with PBS+ 0.1%Tween+ 0.5% bovine serum albumin and incubated with the appropriate secondary antibodies, for 1 h at 37 °C. The coverslips were mounted in a fluorescent mounting medium (DAKO) and imaging was performed on a laser scanning Confocal Microscope Leica (Wetzlar, Germany) SP2 AOBS SE, using the × 40/ × 63 oil objective. Primary antibodies used were anti-GFP (1:250, Santa Cruz, Dallas, TX, USA), anti-megalin (1:200, Biorbyt (Cambridge, UK), orb6173), anti-MAP2 (1:800; Abcam, ab24640), anti-Tau (1:750, Cell Signaling, #4019), as secondary antibodies Alexa Fluor 488 and 594 (1:750, Life technologies) were used. The fluorescent dye Hoechst 33342 (0.5 *μ*g/ml–10 min room temperature) was used to stain nuclei.

### Permanent middle cerebral artery occlusion – pMCAO

Focal cerebral ischemia was induced as described previously.^57^ Male mice were anesthetized subcutaneously with Hypnorm/Stesolid (fentanyl citrate (0.315 mg/ml; Jansen-Cilag, Barcarena, Portugal), Fluanisone (10 mg/ml; Jansen-Cilag) and Diazepamum (5 mg/ml; Dumex, Copenhagen, Denmark)) and placed on a 37 °C heating pad. The left temporoparietal region of the head was shaved and a skin incision was made between the lateral part of the orbit and the external auditory meatus. The superior pole of the parotid gland was pushed downwards as was the upper part of the temporal muscle. A small craniotomy was made with a 0.7-mm burr just over the MCA at the level of the inferior cerebral vein. The inner layer of the skull was removed with fine forceps, and the dura mater opened. The MCA was bipolarly electrocauterized applying forceps coupled to an electrosurgical unit (ICC50; Erbe, Tübingen, Germany). The incision was closed with a 4-0 nylon suture; 1 ml of physiological saline solution (9 g/l) was injected subcutaneously and the eyes coated with ointment to protect from drying. Before killing of the mice, they were kept in a recovery room at 28 °C for 24 h. Post-surgical pain treatment consisted of supplying the mouse subcutaneously with Temgesic (0.001 mg/20 g buprenorphinum; Reckitt & Colman, Hull, UK) every 8 h for the first 24 h. Mice were killed by deeply anesthetizing with pentobarbital. The total number of animals used (all included in the analysis): three animals for each phenotype (WT, TTR KO, both (HSF(+/−) background)) were used for immunohistochemistry and four animals for each phenotype (WT, TTR KO, both (HSF(+/−) background)) for biochemical analysis. Western blot: After sacrifice, the brain was collected and 2 mm brain sections were excised with a 1 mm coronal mouse matrice. The slices were immediately frozen with dry ice and the ipsilateral and contralateral areas of the brain slices were taken using a Harris Unicore 2 mm tip (Pelco International, Redding, CA, USA). Samples were then homogenized and processed for western blot. Histochemistry: After mice were deeply anesthetized, they were transcardially perfused with ice-cold PBS followed by 4% paraformaldehyde in 0.1 M phosphate buffer (pH 7.4). The brains were removed, post-fixed in the same fixative overnight at 4 °C and then left in 30% sucrose in PBS at 4 °C, until sinking in the solution. Coronal sections (20 *μ*m) were cut on a freezing microtome (Leica Cryostat CM 3050) and mounted on gelatin-chromium-covered glass slides. Sections were then used for immunohistochemistry analysis. The experimental unit in both western blot and histochemistry of pMCAO mice was each individual mice (in which the focal cerebral ischemia was performed in separate periods of time, with mice randomly selected in each phenotype (only by age and sex for the total group in experiment)).

### Immunohistochemistry

Slides containing the brain slides from WT and TTR KO (HSF (+/−) background) 24 h pMCAO were removed from freezer and incubated at room temperature for at least 15 min. Then, the slides were incubated with Tris-buffer saline (TBS), and permeabilized with 0.2% Triton X-100 in TBS solution for 10 min and rinsed in TBS 0.025% Triton X-100. Blocking was performed with 10% fetal bovine serum, plus 1% bovine serum albumin and 0.3 M glycine, in TBS, for 2 h at room temperature. Primary antibodies were always incubated overnight at 4 ºC, in TBS 1% bovine serum albumin. Secondary antibodies were incubated 1 h at room temperature. The slides were mounted in a fluorescent mounting medium (DAKO) and imaging was performed on a laser scanning Confocal Microscope Leica SP2 or SP5 AOBS SE, using the × 40/ × 63 oil objective. In each set of experiments, the same batch of antibodies (primary and secondary) was used, and images were taken using the same settings, such as camera exposure times. For the cell survival semi-quantification (Figures 9g and j), cells were counted, that is, either they expressed the indicated epitopes or not. Primary antibodies used were anti-mouse TTR (1:150, Rabbit, custom made), anti-megalin (1:1000, Sheep, custom made), anti-*β*III-tubulin (1:500, monoclonal mouse, Promega, Madison, WI, USA, G712A), anti-BrdU (TUNEL Assay Kit, Abcam, ab66110); as secondary antibodies Alexa Fluor 488 and 568 (1:750, Life Technologies) were used. The fluorescent dye Hoechst 33342 (0.5 *μ*g/ml–15 min room temperature) was used to stain nuclei.

### FRET assay–intracellular calcium concentration

Cells (TTR KO cultured neurons, 80 000 cells/cm^2^) were imaged in poly-D-lysine-coated glass-bottom dishes (ibidi GmbH, Martinsried, Germany) 48 h after transfection with yellow cameleon-Nano15 (YC-Nano15), an ultrasensitive Ca^2+^ FRET probe.^27^ This FRET probe has high calcium affinity, enabling the detection of subtle calcium changes associated with intracellular signaling dynamics and neuronal activity, even at the *in vitro* culture level. Cells were selected for the assay upon two parameters only: transfected with yellow cameleon-Nano15 and the ones that look healthy, with intact neurites. The plasmid was obtained from Addgene (Cambridge, MA, USA) (plasmid #51961). Neuronal cultures were performed using a Neurobasal medium without phenol red (Gibco, Life Technologies) to optimize signal/noise levels. Recombinant TTR stimuli and chemical inhibitors were previously diluted in PBS and applied directly to the conditioned culture medium in the dish. Assays were performed under a humidified and heated chamber, for periods of 20 min. Fluorescence imaging of cells was performed using an epifluorescence inverted microscope (DMI 6000B, Leica Microsystems) with a PlanApo × 63 (N.A. 1.4) glycerol immersion objective. Data acquisition and processing was based on Ferraz-Nogueira *et al.*^58^ Briefly, a mercury lamp coupled to a light attenuator (EL6000, Leica Microsystems) and a bandpass filter was used to excite directly the donor fluorescent protein. The cyan and yellow fluorescence signals were acquired using bandpass filters (480/40 nm and 530/30 nm for cyan and yellow fluorescence, respectively). The setup used a 440/520-nm dichroic mirror (CG1, Leica Microsystems) and the bandpass filters were mounted in external filter wheels (Fast Filter Wheels, Leica Microsystems). The FRET/donor change was calculated using ImageJ software. Customized macros were used to subtract the background from raw images and to create intensity-modulated ratio images. The time axis in the plots was adjusted to consider the TTR administration time as '0 s' (dashed line in the graphs), but first time analyzed after stimulation is '0.33 s'. Drugs and inhibitors used in this FRET assay were always pre-incubated for 15 min before TTR stimulation: SKI (200 nM), kynurenic acid (2 mM, Sigma) and MK801 (50 *μ*M, Calbiochem, San Diego, CA, USA). NMDA (10 *μ*M, Sigma) was used to stimulate neurons, that is, NMDA receptors. The experiments performed without calcium or with 60 mM of KCl used a Krebs-Ringer solution. The composition of the solution used in the experiment with KCl consisted of: 119 mM NaCl, 60 mM KCl, 1.0 mM NaH_2_PO_4_, 2.5 mM CaCl_2_.2H_2_O, 1.3 mM MgCl_2_.6H_2_0, 20 mM HEPES and 11 mM D-glucose, pH 7.4. The solution of the experiment without calcium had the same composition with the exception of KCl, which was present at a smaller concentration (2.5 mM), and CaCl_2_, which was replaced with 2 mM EGTA. The experimental unit in FRET assays was each individual neuron (four to six neurons were analyzed in each individual culture).

### Statistical analysis

Protein values were normalized for *α*-tubulin or non-phosphorylated forms of the protein in study, whereas mRNA transcripts were normalized for 18 S mRNA. Data are presented as mean±S.E.M. of at least three different experiments (exact number described in figure legends), performed in independent preparations. A previous power analysis was performed in order to obtain a 25% difference (with 10% S.D.) among two groups, with 90–95% power and we obtain sample sizes of three to six animals or individual cultures. Statistical analysis of the results was performed using one-way analysis of variance (ANOVA) followed by Bonferroni multiple comparison test, when three groups were present: ***P<0.001, **P<0.01, *P<0.05, n.s. (not significant). Unpaired Student's *t*-test was used when the comparisons were only between two groups: ***P<0.001, **P<0.01, *P<0.05, n.s. (not significant).

## Abbreviations

TTR: transthyretin
pMCAO: permanent middle cerebral artery occlusion
CREB: cAMP response element-binding protein
Bcl2: B-cell lymphoma 2
NMDA: N-methyl-D-aspartate
NR2A: N-methyl-D-aspartate 2A receptor
