# Supplementary figures and images for: Transthyretin provides trophic support via megalin by promoting neurite outgrowth and neuroprotection in cerebral ischemia
Source: Cell Death Differ. 2016 Aug 12;23(11):1749–64. doi: 10.1038/cdd.2016.64 (PMC5071567; doi:10.1038/cdd.2016.64)

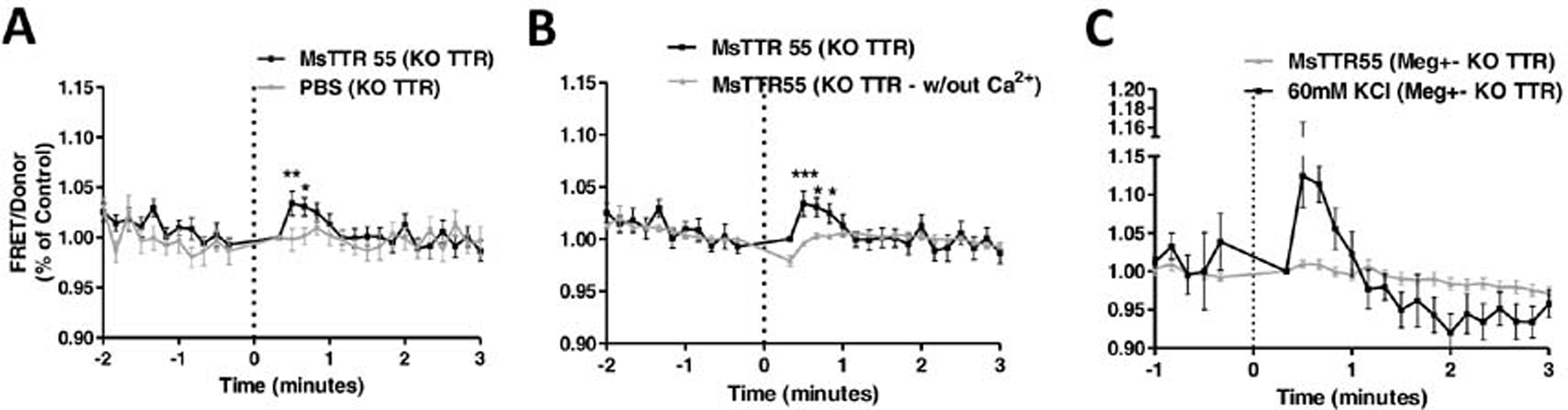

Supplement: Supplementary Figure 1 [file cdd201664x1.tif]

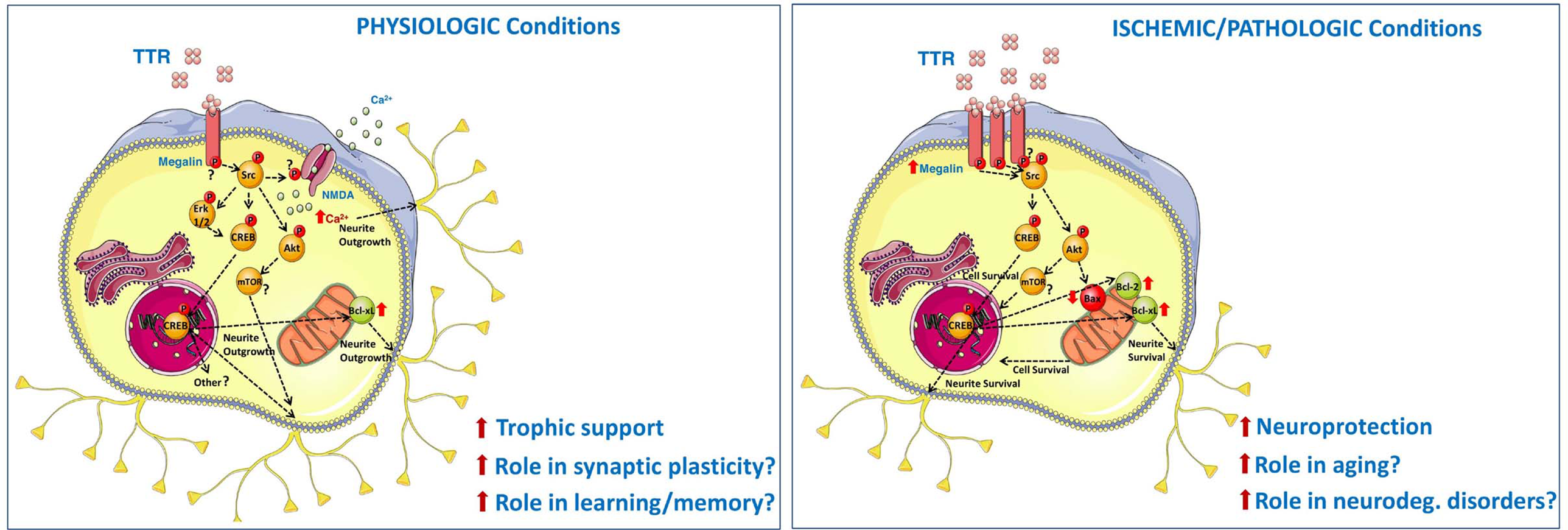

Supplement: Supplementary Figure 2 [file cdd201664x2.tif]
