# Supplementary material for: Transthyretin provides trophic support via megalin by promoting neurite outgrowth and neuroprotection in cerebral ischemia
Source: Cell Death Differ. 2016 Aug 12;23(11):1749–64. doi: 10.1038/cdd.2016.64 (PMC5071567; doi:10.1038/cdd.2016.64)
Supplement: Supplementary Figure Legend [file cdd201664x3.docx]

**Fig. S1- Complementary FRET assays.** A, Time course of normalized FRET/Donor values in cell body of YC-Nano15 transfected TTR KO cultured hippocampal neurons (7DIV) stimulated with TTR (55µg/ml), compared to the corresponding volume of PBS (MsTTR, data from Fig. 2F; PBS, 4 independent experiments, 5-6 neurons in each experiment). (B), Same experimental design as in (A), but cultures were challenged with 55µg/ml of mouse TTR in a culture medium with or without calcium. (for MsTTR, data from Fig. 2F; for MsTTR without calcium, 2 independent experiments, 6 neurons in each experiment). (C), Time course of normalized FRET/Donor values in cell body of YC-Nano15 transfected Megalin (+/-) TTR KO cultured hippocampal neurons stimulated with mouse TTR (55µg/ml) and a depolarizing 60mM KCl culture medium (for MsTTR, data from Fig. 2O and for KCL, 1 independent experiment, 5 neurons). Statistical analysis was performed using One Way ANOVA followed by Bonferroni’s multiple comparison test performed for each. ***p<0.001, **p<0.01, *p<0.05, n.s.- not significant as compared to the control or as indicated.

**Fig.S2- Proposed mechanism of TTR neurotrophic action in physiologic and ischemic conditions.** Cartoon showing the effect of TTR, mediated by Megalin (LRP2) receptor in neuronal populations in the CNS, either in physiologic or pathological conditions (excitotoxicity and cerebral ischemia). TTR is able to promote neurite outgrowth in physiological conditions, in a Megalin dependent way. It will activate Src, which in turn will activate NMDA receptors, probably through phosphorylation, allowing a transient rise in intracellular calcium. Moreover ERK1/2, Akt and CREB will also be activated by Src promoting an increase in the neurite number and length. mTOR, an Akt target, and BclXL might also be involved in this neurite outgrowth as suggested by the literature [^59^](#_ENREF_59)^,^ [^60^](#_ENREF_60). Under pathological conditions, like excitotoxicity and cerebral ischemia (pMCAO), TTR is also able to be neuroprotective in two ways, by promoting neuronal survival and by preserving more neurites after the excitotoxic insult. TTR action is mediated by Akt and CREB, which will regulate the balance between anti-apoptotic (Bcl2 and BclXL) and pro-apoptotic proteins (Bax), towards survival. BclXL and mTOR, according to the literature, might also have an important role in neurite survival and cell survival, respectively [^59^](#_ENREF_59).
